# Influence of Porcine Intervertebral Disc Matrix on Stem Cell Differentiation

**DOI:** 10.3390/jfb2030155

**Published:** 2011-08-08

**Authors:** Denise Salzig, Alexandra Schmiermund, Elke Gebauer, Hans-Lothar Fuchsbauer, Peter Czermak

**Affiliations:** 1 Institute of Bioprocess Engineering and Pharmaceutical Technology, University of Applied Sciences Mittelhessen, Wiesenstraße 14, 35390 Giessen, Germany; E-Mails: alexandra.schmiermund@kmub.th-mittelhessen.de (A.S.); peter.czermak@kmub.th-mittelhessen.de (P.C.); 2 Department of Chemical Engineering and Biotechnology, University of Applied Sciences, 64297 Darmstadt, Germany; E-Mails: gebauer-elke@gmx.de (E.G.); fuchsbauer@h-da.de (H.-L.F.); 3 Department of Chemical Engineering, Kansas State University, Manhattan, KS 66506, USA

**Keywords:** nucleus pulposus extract, gelatin, mesenchymal stem cells, transglutaminase

## Abstract

For back disorders, cell therapy is one approach for a real regeneration of a degenerated nucleus pulposus. Human mesenchymal stem cells (hMSC) could be differentiated into nucleus pulposus (NP)-like cells and used for cell therapy. Therefore it is necessary to find a suitable biocompatible matrix, which supports differentiation. It could be shown that a differentiation of hMSC in a microbial transglutaminase cross-linked gelatin matrix is possible, but resulted in a more chondrocyte-like cell type. The addition of porcine NP extract to the gelatin matrix caused a differentiation closer to the desired NP cell phenotype. This concludes that a hydrogel containing NP extract without any other supplements could be suitable for differentiation of hMSCs into NP cells. The NP extract itself can be cross-linked by transglutaminase to build a hydrogel free of NP atypical substrates. As shown by side-specific biotinylation, the NP extract contains molecules with free glutamine and lysine residues available for the transglutaminase.

## 1. Introduction

Back disorders affect many people in industrialized countries and thus are a large economic problem. These medical conditions can be caused by the degeneration of the intervertebral disc (IVD), particularly the nucleus pulposus (NP). An alternative to the often complicated surgical treatments of the disc would be a real regeneration of the IVD by cell treatment with NP-cells or with into NP-cells pre-differentiated human mesenchymal stem cells (hMSCs). In contrast to NP cells, these pluripotent stem cells have been found in almost every organ in adulthood. These cells are of high plasticity and have the capacity of multilineage differentiation. In addition, they are accessible in sufficient quantities from bone marrow and fat tissue and comparably easy to expand and manipulate which make them ideal candidates for cell-based IVD regeneration [1].

For the differentiation in NP cells and to maintain this specific phenotype and genotype, hMSCs need a three-dimensional environment [2]. In addition to the three-dimensionality, the composition of the matrix and the environmental parameters itself exert a strong stimulus to the cell maintenance and differentiation.

### 1.1. Differentiation of Progenitor Cells in Nucleus Pulposus Cells

From an evolutionary perspective, hMSCs are not the natural progenitor cells of NP cells. During embryonic development, the NP cells are made of notochordal cells. However, the notochordal cells regress relatively quickly after birth, so that in adult humans these cells cannot be used for regeneration of the NP [3]. Compared to that, hMSCs are present lifelong in sufficient number and show a high potential for disc regeneration *in vivo* [4]. In addition to autologous hMSCs, allogenic cells can also be used in the treatment of the NP. Advantages of allogenic hMSCs are off-the-shelf availability which omits the time span for cell isolation and expansion. Moreover, the use of allogeneic MSCs eliminates potential genetic dispositions and limited potency depending on the age of the patient. Therefore in the current study an allogenic cell line (hMSC-TERT) was investigated.

*In vitro*, hMSCs differentiate into NP-like cell type with the addition of growth factors (e.g., TGF-β 3) or by direct cell contact with NP cells [5,6]. Also, oxygen deficiency (hypoxia) promotes the differentiation of mesenchymal stem cells to NP-like cells [7]. It has been known for several years that the matrix exerts a strong influence on cell differentiation as well. Nevertheless, for the differentiation of hMSCs into NP cells, this influence has never been investigated.

### 1.2. Extracellular Matrix of Nucleus Pulposus

In contrast to cartilage or annulus fibrosus (outer region of IVD), the extracellular matrix (ECM) of the NP is characterized by a high type II collagen and proteoglycan (PG) content. The main components of the ECM are PGs which are present as monomers. During degeneration, the matrix composition is modified due to changed expression profiles of the cells. Instead of type II and IX collagen, mainly type I, III and X collagen are produced. Simultaneously, the production of PGs decrease [8]. The beginning of the NP degeneration process is determined by this matrix changes. This process can hardly be reversed in the human body by itself. Treatment with healthy cells (diff hMSCs or NP cells) in an appropriate biomaterial is a promising possibility to restore NP functionality.

### 1.3. Biomaterials for NP Regeneration

In the past, sponge-like or ceramic materials have been favored as biomaterials for tissue regeneration. Later, it turned out that the biomaterial should behave as similarly as possible to the natural ECM to obtain cell phenotypes that correspond to the *in vivo* situation. In the case of the NP, this is realized mostly by hydrogels [9]. In tissue regeneration, following biopolymers are often used: agarose, alginate, collagen, fibrin, gelatin and hyaluronic acid [9,10,11]. Agarose and alginate have the disadvantage that they are not biodegradable, and cell adhesion is often low. The others are both biodegradable and highly adhesive for most cells. These and other materials have been investigated in the past for their suitability for in vitro or in vivo NP-regeneration.

Different cell types (IVD cells, NP cells, AF cells, MSCs) from various species were embedded and analyzed for these experiments. Unfortunately, the comparability between studies is often impossible because the experimental setup was different. Many materials had a general beneficial effect on the survival and matrix synthesis of NP cells or IVD cells. MSCs were also successfully proliferated and differentiated into NP-like cells in some materials (e.g., atelocollagen, alginate, hyaluronic acid, PLLA) [12,13]. An *in vitro* investigation of the effect of gelatin or NP extract on the differentiation capacity of hMSCs into NP-like cells is not published yet.

### 1.4. Aim of the Study

Former studies investigated the influence of different growth factors on the differentiation of in agarose embedded hMSCs towards NP cells [6]. The current study investigates the influence of different matrices on mesenchymal stem cell differentiation. The embedding material used in these experiments was gelatin. Gelatin was used as model protein, because it can be easily cross-linked via transglutaminase. Bacterial transglutaminase was produced in a well characterized procedure [14]. The first aim of the study was to analyze the differentiation behavior of in gelatin embedded hMSCs. Gelatin consists of type I collagen fibers. We were concerned whether this matrix influences the differentiation of hMSCs compared to an inert matrix like agarose. Secondly, the effect of porcine NP extract should be investigated. It is known that hMSCs differentiate in direct contact with NP cells towards the NP lineage [5]. This study wanted to clarify whether the matrix itself is adequate to induce differentiation into NP cells. Further, the NP extract was purified and prepared for cross-linking with transglutaminase. The aim was to prepare a hydrogel which only consists of natural NP material.

## 2. Experimental Section

### 2.1. Isolation of Porcine Nucleus Pulposus Extract

Porcine nucleus pulposus was collected from juvenile pigs one day after slaughtering from a local abattoir and frozen at −80 °C.

Thawed NP was washed twice with Milli Q water and extracted by using 0.15 M NaCl in 2 mM phosphate buffer pH 7.4 (5 mL/g) under gentle stirring for two days at 4 °C.

### 2.2. Production and Purification of the Transglutaminase

Microbial transglutaminase (TGase) was prepared from cell-free culture supernatants of *Streptomyces mobaraensis* (strain 40847, DSMZ, Brunswick, Germany) as described previously.

### 2.3. Transglutaminase Mediated Glutamine and Lysine Biotinylation of Nucleus Pulposus Extract

**Figure 1:**
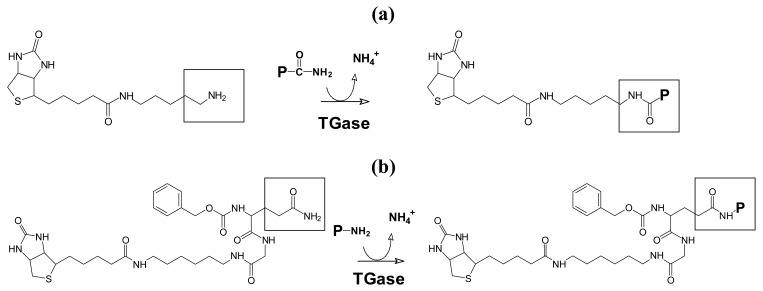
(**a**) Transglutaminase mediated biotinylation of glutamine; (**b**) Transglutaminase mediated biotinylation of lysine donor proteins.

Cross-linking sites of proteins in the NP extract were determined by transglutaminase mediated incorporation of monobiotinylcadaverine (probe for glutamine residues) and 1-N-biotinyl-6-N′-(carbobenzoxy-L-glutaminylglycyl)hexanediamine (probe for lysine residues) as described [15] (Figure 1). The biotinylated compounds were purchased from Pierce (Rockford, IL, USA) and Zedira (Darmstadt, Germany).

### 2.4. Preparation and Cultivation of the Cell-Matrix-Hydrogel

For all experiments, a hMSC cell line (hMSC-TERT) was used [16]. For cell expansion, hMSC-TERT were cultivated and harvested according to standard procedures as described before [17,18].

For the hydrogel preparation, 7% or 12.5% (w/v) gelatin (porcine, typA, 300 g Bloom; Gelita, Eberbach, Germany) was allowed to swell for 30 min in phosphate buffered saline (PBS) (without Ca^2+^ and Mg^2+^) (PAA, Austria) or NP extract (2.1). The mixture was gently heated up until the gelatin was completely dissolved. The solution was sterilized by filtration through a 0.2 μm syringe filter (Carl Roth, Karlsruhe, Germany). Lyophilized TGase (2.2) was dissolved in 50 mM TRIS-acetat-buffer (pH 6) to a final concentration of 9.6 U/mL. hMSC-TERT were harvested and resuspended in differentiation medium with a density of 1.35 × 10^7^ cells/cm^3^. Differentiation media consisted of Dulbecco's modified Eagle's medium (DMEM-LG; Biochrom, Berlin, Germany) containing 3.7 mg/mL NaHCO_3_ (Carl Roth, Karlsruhe, Germany), 100 U/mL Penicillin and 100 μg/mL Streptomycin (both Biochrom), 1× Insulin-Transferrin-Selenium-X (Invitrogen, Darmstadt, Germany), 0.5 mg/mL bovine serum albumin (Carl Roth), 1.25 mg/mL human serum albumin (Sigma-Aldrich, Steinheim, Germany), 4.7 μg/mL linolic acid (AppliChem, Darmstadt, Germany), 0.1 μM dexamethasone (Sigma-Aldrich), 0.1 mM 2-phospho-L-ascorbic acid trisodium salt (Sigma-Aldrich) with or without 0.28 nM TGF-β3 (Biochrom).

A cylindrical hydrogel was prepared at 37 °C by mixing stock solutions of TGase (25 μL), gelatin (136 μL) and cells (68 mL) resulting in hydrogels with a cell density of 4 × 10^6^ cells/cm^3^ (corresponding to the natural cell density in the NP). The cell-matrix-hydrogels were cultivated for 21 d in humidified CO_2_ atmosphere using 24-well-plates containing differentiation medium. Medium was exchanged every three days.

### 2.5. Live-Dead-Staining of Cells

Cell survival was estimated by SybrGreenI (SG; 10,000×, Sigma-Aldrich) and propidium iodide (PI; 1g/L, Carl Roth) staining. Therefore, the two reagents were mixed, diluted with PBS (Biochrom) to final concentration of 7× (SG) or 0.2 g/L (PI). After addition to the hydrogel for 15 min, SybrGreen/PI stained hydrogels were transferred into a multiwell plate with glass bottom and kept in PBS to prevent dehydration. The fluorescence of the dyes was analyzed by fluorescence wide-field microscopy fluorescence (DMI6000, Leica Microsystems, Wetzlar, Germany) using filter cubes L5 (ex.: BP 480/40, em.: BP 527/30) and N3 (ex.: BP 546/12, em.: BP 600/40) and LAS AF Software (Leica Microsystems).

### 2.6. Measurement of Cell Proliferation

To determine metabolic cell activity at various times, WST-1 reagent (Roche Diagnostics, Mannheim, Germany) was used. A working solution was made by diluting WST-1 in a ratio of 1:100 in differentiation medium. 1 mL of this solution was added to the cell-matrix-hydrogels and was incubated for 20 h at 37 °C in a humidified CO_2_ atmosphere. Differentiation medium with WST-1 alone was the negative control. 100 μL of the incubated medium were transferred into a 96-well-plate, and the absorbance at 450 nm was measured with a microplate reader (Synergy HT, BioTek, Bad Friedrichshall, Germany).

### 2.7. Expression of Specific Marker Genes (RT-PCR)

Total RNA was extracted from embedded cells using QIAzol Reagent (Qiagen GmbH, Hilden, Germany) [19]. cDNA was prepared using the Omniscript kit (Qiagen GmbH) according to the manufacturer's instructions. PCR was performed using the TaqCore kit (Qiagen GmbH) and gene specific primers (Table1). As reference, the housekeeping gene glyceraldehyde-3-phosphate dehydrogenase (GAPDH) was chosen.

**Table 1:** Oligonucleotide primers for RT-PCR amplification. For analysis, genes with high expression in human chondrocytes or human nucleus, pulposus cells were chosen. The primers were made by using Primer3 Software [20] and are based on the reference sequences of human mRNA of the genes. The mRNA was characterized over the GI (GenInfo Indentifier) number.

**Gene Name**	**Gene Symbol**	**Reference Sequence (GI)**	**Forward Primer (5′-3′)**	**Reverse Primer (5′-3′)**
***housekeeping gene***				
glyceraldehyde-3-phosphate dehydrogenase	GAPDH	83641890	AATCAAGTGGGG CGATGCTGGCGCT	AGTGTGGCAGGG ACTCCCCAGCAGT
***genes coding for matrix proteins***				
type I collagen	COL1A1	110349771	GGGCCTCAGGGT GCTCGAGGATTC	AGGGCTGCCAGG GCTTCCAGTCAGA
type II collagen	COL2A1	111118973	TGACGGTCCCTCT GGTGCCGAAGGT	CGGGGCCCTTCT CTCTCGGGCCTAA
type X collagen	COL10A1	98985802	ACCCACAGGAGC CCCAGGAC	GCTATGCCAGCT GGGCCAGG
versican	VCAN	255918075	CCCACCGGTGAG GGGCTCCCT	GGTGCCTCCGTT AAGGCACGGGT
aggrecan	ACAN	223462184	CGCCGGTGTCGG GAGCAGCA	TGCGTTTGTAGGT GGTGGCTGTGC
***genes coding for putative chondrocyte markers***				
cartilage oligomeric protein	COMP	40217842	ACTGCAGGAAAC CAACGCGGCGC	GGTTCCGCACCA GCGGGCAGTT
integrin-binding sialoprotein	IBSP	167466186	AGGGCAAGGGCA CCTCGAAGA	TCATTGGCGCCC GTGTATTCGT
***genes coding for putative NP cell markers***				
neural cell adhesion molecule 1	NCAM1	117320546	GGCTTCGTGGAC TCGACCAGAG	TAGTGTCTGATG GGGGAGCCGC
annexin A3	ANXA3	96304463	GGCGCGGGAACA AACGAAGATGCC	GGCCGGCGTGTT CCTCACACAA
pleiotrophin	PTN	42476152	GGGCACACGGGA GGGCACTCG	TGGTCAGTTTGCC ACAGGGCTTGGA
glypican 3	GPC3	257471005	GCCGCCGGACGC CACCTGTC	GGCTGAATCAGG CAGGCCTGGGT
brachyury	T	2558580	AGCTCCCCTGGC ACCGAGAG	GCGTGGAGGGGA GGGAGAGG
paired box 1	PAX1	153791841	TGGCGCGCTACA ACGAGACC	GCGCCAGAGGAG GACCTTGC

The results of the RT-PCR experiments were analyzed via horizontal gel electrophoresis according to the manufactures' protocol.

## 3. Results and Discussion

The suitability of gelatin to be cross-linked by microbial transglutaminase (TGase) is well described. Therefore, in this study, gelatin was used as model protein. The first aim of the study was to investigate whether a differentiation of hMSC-TERT in TGase cross-lined gelatin is possible and if the cells develop an NP-like cell phenotype when stimulated with the growth factor TGF-β3 according to the standard differentiation protocol. Furthermore, it was of interest to investigate whether porcine NP extract improves the differentiation behavior when added to the gelatin matrix.

### 3.1. Cell Survival and Proliferation of in Gelatin Embedded and Differentiated hMSC-TERT

hMSC-TERT cells were embedded in gelatin, cross-linked by the addition of TGase and differentiated as described. Three hydrogel compositions were investigated: (1) 7% (w/v) gelatin, (2) 12.5% (w/v) gelatin and (3) a mixture of 12.5% (w/v) gelatin with NP extract. Moreover, differentiation of hMSC-TERT in hydrogel composition 3 was investigated in differentiation medium with and without growth factor TGF-β3.

**Figure 2:**
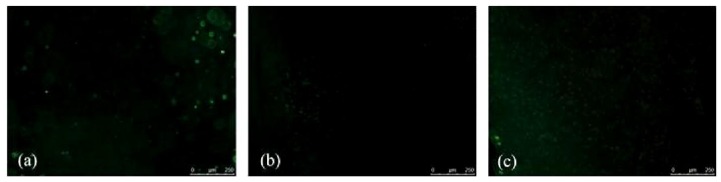
Live-dead-staining of in gelatin embedded and differentiated hMSC-TERT cells. hMSC-TERT were embedded in (**a**) 12.5% (w/v) gelatin, (**b**) and (**c**) a mixture of 12.5% (w/v) gelatin and NP extract, cross-linked by microbial TGase and cultivated for 21 days in differentiation medium with TGF-β3 (**a**,**b**) or without TGF-β3 (**c**). Embedded cells were stained (SybrGreen/PI) and analyzed in a fluorescence microscope using a HCX FL PLAN 10×/0.25 objective (Leica Microsystems, Germany).

In all approaches, cells survived the whole culture period. Figure 2 shows the live-dead-staining of hMSC-TERT differentiated in 12.5% (w/v) gelatin for 21 days. Almost no dead (red) cells were visible even after 21 days culture indicating a cell survival of approximately 100%. Live-dead-stained cells in 7% (w/v) gelatin resulted in a similar image as in Figure 2 (data not shown).

Beside cell survival, cell proliferation was analyzed over 21 days. In all approaches, cell activity was seen the whole differentiation time. There were no differences between the diverse attempts. Furthermore, cell proliferation did not increase during differentiation time. There are only few data about the proliferation of hMSCs in gelatin. In gelatin/PLLA sponges hMSC adhered and proliferated [21,22]. NP cells proliferated in gelatin (without enclosed TGase) as well and showed no increase in cell activity during two and four week's cultivation [23].

These experiments showed that hMSC-TERT survive and proliferate in TGase cross-linked gelatin, independently from the gelatin concentration, the availability of nucleus pulposus extract or TGF-β3.

### 3.2. Morphological Changes after Differentiation of hMSC-TERT in Gelatin

**Figure 3:**
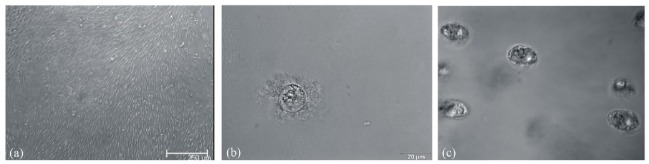
Phenotype of (**a**) undifferentiated hMSC-TERT, (**b**) human nucleus pulposus cells and (**c**) in cross-linked gelatin differentiated hMSC-TERT cells. hMSC-TERT were embedded in 12.5% (w/v) gelatin, crosslinked by TGase and cultivated for 21 days in EMEM medium. The NP cells (#4800; ScienCell, Carlsbad, USA) were embedded in 2% (w/v) agarose and cultivated for 21 d in NP cell medium (#4801, ScienCell). hMSC-TERT were embedded in 12.5% (w/v) gelatin, crosslinked by TGase and cultivated for 21 d in differentiation medium. Embedded cells were analyzed in a bright field microscope using UPlanSApo 60x, 1.2 WI objective (Olympus; Hamburg, Germany).

Morphological investigation of in cross-linked gelatin differentiated hMSC-TERT yielded the same phenotype for all approaches. Cells were not rounded and surrounded by extracellular matrix as typical for NP cells (Figure 3b). Their shape was more elliptical, and the cells lay in lacunas (Figure 3c). This morphology is typical for chondrocytes. Based on the phenotype images, it is obvious that the stem cells differentiated. Undifferentiated hMSC-TERT have a fibroblastic phenotype even in a hydrogel (Figure 3a). Nevertheless, no changes were visible between the various differentiation approaches. Furthermore, it appears that the cells differentiated into chondrocytes instead of NP cells.

### 3.4. Gene Expression during hMSC-TERT Differentiation in Gelatin

To have a deeper insight in the hMSC-TERT differentiation in these four approaches, RT-PCR was performed. Based on literature data, genes were selected which expression are upregulated in chondrocytes or NP cells. Studies in the literature have concentrated on a comparison of NP cells and chondrocytes from animal or human models. Fujita *et al.* compared the expression pattern of eight rat tissues. GPC3 was one of 10,490 genes which was highly expressed in NP [24]. Lee *et al.* discovered that the mRNA of ANXA3, GPC3 and PTN were upregulated in NP cells from rats. Further COMP was expressed in higher rates in chondrocytes [25]. Sakai *et al.* observed that expression rate of e.g., NCAM1 was higher in canine NP cells [26]. Rutges *et al.* investigated human cells from various patients. In this case, the mRNA levels of NCAM1 were significantly higher in NP cells [27]. Minogue *et al.* identified highly expressed IBSP in bovine/human chondrocytes, and upregulated PAX1 and T in human and bovine NP cells, respectively [28,29]. Collectively, it can be said that all markers found in NP cells are, though in other quantities, also expressed in chondrocytes. Moreover, little is known about their expression in hMSCs. Concerning their matrix, chondrocytes and NP cells differ. Depending on the specific type, the ECM of chondrocytes has high amounts of type I, II and X collagen and of large proteoglycans (e.g., aggrecan) [30]. NP cells express mainly type II collagen and smaller and more monomeric proteoglycans (e.g., versican) [31].

Based on these investigations several putative NP marker genes (ANAX3, GPC3, NCAM1, PAX1, PTN and T), putative chondrocyte marker genes (COMP, ISBP) and genes encoding for matrix proteins (COL1A, COL2A, COL10A, ACAN and VCAN) were analyzed in the current study. Table 2 shows the expression profile of in cross-linked gelatin embedded and differentiated hMSC-TERT cells.

**Table 2:** Summary of the RT-PCR results for in cross-linked gelatin embedded and differentiated hMSC-TERT cells. hMSC-TERT were embedded in 7% (w/v) gelatin, 12.5% (w/v) gelatin or in a mixture of 12.5% (w/v) gelatin and NP extract, cross-linked by TGase and cultivated for 21 d in differentiation medium with TGF-β3 or without TGF-β3. Undifferentiated hMSC-TERT were the control (NC). RNA of the cells was isolated and RT-PCR was performed as described in materials and methods. The presence of GAPDH shows the functionality of the RT-PCR. Gray boxes symbolize the expression of the gene at the indicated time, white boxes the absence of gene expression (n = 3).

		**NC**	**7% (w/v) gelatin** **-NP extract** **+TGF-b3**						**12.5% (w/v) gelatin** **-NP extract** **+TGF-b3**						**12.5% (w/v) gelatin** **+NP extract** **+TGF-b3**						**12.5% (w/v) gelatin** **+NP extract** **-TGF-b**					
	**differentiation time [d]**	**-**	**4**	**7**	**11**	**14**	**17**	**21**	**4**	**7**	**11**	**14**	**17**	**21**	**4**	**7**	**11**	**14**	**17**	**21**	**4**	**7**	**11**	**14**	**17**	**21**
	**gene symbol**																									
**putative NP cell marker**	ANAX3																									
	GPC3																									
	NCAM1																									
	PAX1																									
	PTN																									
	T																									
**putative chrondrocyte marker**	COMP																									
	IBSP																									
**matrix proteins**	COL1A1																									
	COL2A1																									
	COL10A1																									
	ACAN																									
	VCAN																									
**housekeeping gene**	GAPDH																									

In all hydrogel compositions, COL1A1, COL10A1, IBSP and PTN were expressed through the whole differentiation period. In undifferentiated hMSC-TERT, COL1A and PTN were expressed as well. Therefore, these two genes are not suitable as marker gene in a RT-PCR experiment and are not discussed below. The expression of COL10A1 indicates the development of hypertrophic cartilage (precursor of bone). In most cellular approaches for cartilage or IVD regeneration using cells expressing of type X collagen will be avoided, because it has been shown to be intimately associated with the calcification process [32]. IBSP is a major structural protein of the bone matrix. It is synthesized by skeletal-associated cell types, including hypertrophic chondrocytes, osteoblasts, osteocytes, and osteoclasts [33]. In literature it is described as a human NP negative marker [28]. This sustains the morphological observations that in all approaches the developed phenotype was basically a chondrocyte-like one. Furthermore, the presence of these two genes could even imply a hypertrophic chondrocyte development.

#### 3.4.1. Influence of Gelatin Concentration

The differentiation approaches with hMSC-TERT in a cross-linked matrix of 7% or 12.5% (w/v) gelatin showed a similar expression pattern for COL2A1, COMP, GPC3 and PAX1. GPC3 was expressed over the full differentiation period. GPC3 was found to be highly expressed in rat NP cells, but only low in human NP cells [29]. It encodes for a protein involved in cell division, growth regulation and apoptosis [34]. During differentiation, COL2A1 was expressed from day 17 to day 21 and COMP from day 11/14 until the end of the cultivation. PAX1 was not found at any time in these two attempts. Whereas COMP and COL21A are both NP and chondrocyte markers, PAX1 is really upregulated in human NP cells. PAX1 is a transcription factor which regulates pattern formation during embryogenesis [35]. Its role in the NP still needs to be clarified. The complete absence of PAX1 supports the hypothesis that in cross-linked gelatin differentiated hMSC-TERT developed a chrondrogenic rather than a NP cell type.

Differences were found in the expression of ACAN, ANXA3, NCAM1, T and VCAN. ACAN was only expressed by cells in the 12.5% (w/v) gelatin matrix approach and NCAM1 only in the 7% (w/v) gelatin until day 7. ACAN is typically present in the ECM of chondrocytes and NP cells. NCAM1 is an adhesion molecule important for cell-cell and cell-matrix interactions. It is found during the early stages of chondrogenesis but does not seem to be specific for chondrocyte differentiation [36]. ANXA3 was expressed only in case of 7% (w/v) gelatin until day 7 whereas expression in the 12.5% (w/v) gelatin approach started at day 14 until the end of cultivation. ANXA3 is only low expressed in human NP cells, but high in murine ones [29]. It is important for cell growth regulation and plays a role in certain signal transduction pathways. T was expressed almost during the whole cultivation of hMSC-TERT in 12.5% (w/v) cross-linked gelatin while for cells in a less dense hydrogel of 7% (w/v) gelatin expression occurred in a later differentiation phase. T encoded for a transcription factor which effect the transcription of genes required for mesoderm formation and differentiation. Furthermore, this protein is assigned to notochord-derived cells [37]. The presence of T could be a hint that there is differentiation in NP cells. VCAN was determined over the whole time in cells grown in the 12.5% (w/v) gelatin, but not in the 7% (w/v) gelatin, displaying a gap (days 7, 11 and 14) within the differentiation period. VCAN is a component of the early matrix in chondrocyte differentiation. Because quantitative measurements are missing, it is hardly possible to make a statement about the influence of gelatin density on hMSC-TERT differentiation. In both cases a chondrogenic phenotype seemed to be developed without a direct link towards the NP linage.

#### 3.4.2. Influence of Nucleus Pulposus Extract

hMSC-TERT differentiation (in 12.5% (w/v) cross-linked gelatin) with and without NP extract yielded in a similar expression of COMP, T and VCAN. T and VCAN were present almost the whole differentiation period while the expression of COMP started at day 14 in both approaches.

Lots of genes were expressed earlier (COL2A1, ANXA3) or exclusively (NCAM1, PAX1) in the approach with NP extract. Expression of GPC3 was down-regulated during differentiation of hMSC-TERT with NP extract. ACAN was only present in the approach without NP extract. It is highly probable that, the NP extract influenced the differentiation of hMSC-TERT. Although the cell phenotype was still chondrogenic, the NP extract induced the onset of differentiation earlier. Moreover, only with NP extract, the putative NP cell marker PAX1 was expressed, strongly indicating a development towards the NP linage.

#### 3.4.3. Influence of TGF-β3

hMSC-TERT embedded in 12.5% (w/v) gelatin with NP extract were cultivated with and without TGF-β3. The results of the two experimental groups showed similar expression of ACAN, ANXA3, GPC3 and T. While expression of ACAN was missing in both cases, ANXA3 was displayed over almost the entire period. GPC3 was only expressed at days 4 and 7. An expression gap of T occurred in the middle of the differentiation so that coding mRNA was only determined at the beginning and the end of cultivation.

Transcription of PAX1 in differentiating hMSC-TERT was only observed by addition of NP extract and TGF-β3. Expression of COL2A1, COMP and VCAN was earlier visible in cells cultivated with TGF-β3. Expression of NCAM1 completely differs between these two approaches. With TGF-β3, NCAM1 was expressed at the beginning of the differentiation whereas, without TGF-β3, the mRNA could be only detected at the end. Without TGF-β3, a differentiation of the hMSC-TERT appeared possible but was significantly slowed down. This was indicated by the late expression of COL2A1, COMP, NCAM1 and VCAN. PAX1 was not expressed in cells cultivated without TGF-β3. Therefore a differentiation of hMSC-TERT into NP cells could require TGF-β3 in certain amounts.

### 3.5. Determination of Transglutaminase Substrates in Nucleus Pulposus

As shown above, NP extract in cross-linked gelatin matrix is superior for the differentiation of hMSC-TERT into NP-cells. Nevertheless, the influence of the gelatin seems to be so strong that a chondrogenic phenotype was expressed at the end, independently from the stimulus. Therefore it would be of great interest to build a gelatin-free hydrogel based on NP components. With such a hydrogel, the influence of NP extract could be investigated independently from the strong influence of a type I collagen hydrolysate (gelatin). To form a hydrogel from NP extract, the components must be suitable for cross-linking with e.g., TGase. NP extract contains type II collagen which could be a substrate for crosslinking via TGase. In the current study, NP extract and type II collagen were investigated for putative substrates of bacterial TGase.

For this purpose, the jelly-like nucleus pulposus of intervertebral discs was scraped from the backbone of halved pigs, one-day after slaughtering, using fumigated spatulas and scalpels and stored at −80 °C. An extract was made by gently stirring the thawed, multiply water-washed raw material in phosphate buffer (pH 7.4) containing 10 mM EDTA and 0.15 M NaCl for two days at 4 °C, using a modified procedure of Aladin *et al.* (2010) [38]. After removal of the remaining solids by centrifugation, the supernatant was dialyzed threefold against 1 mM EDTA in TRIS acetate buffer (pH 6). The dialyzed extract was treated with monobiotinylcadaverine or the biotinylated glutamine dipeptide ZQGB to display glutamine and lysine donor proteins, respectively (Figure 1). NP extract without TGase and pepsin-solubilized bovine type II collagen were the controls.

**Figure 4:**
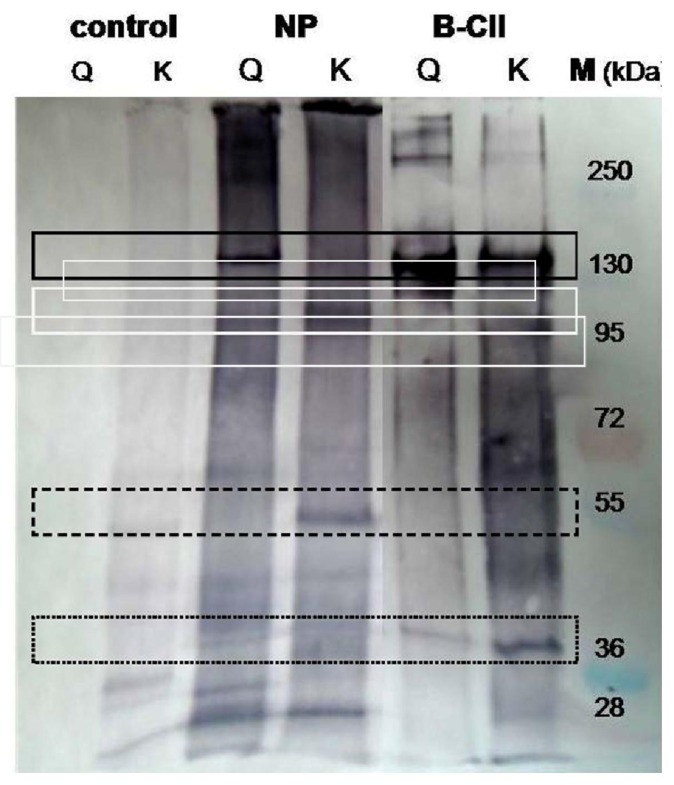
Biotinylation of porcine nucleus pulposus proteins by transglutaminase mediated incorporation of glutamine and lysine probes. Extract of nucleus pulposus in 100 mM HEPES buffer (pH 7) and 2 mM monobiotinylcadaverine (probe for accessible glutamine residues, lines Q) or 0.13 mM of a biotinylated glutamine dipeptide (probe for accessible lysine residues, lines K) were incubated with 1 U/mL TGase at 37 °C for 1.5 h (lines NP). For comparison, extract of nucleus pulposus without TGase (control) and purified bovine type II collagen (B-CII) were used. Staining was performed using streptavidin alkaline phosphatase conjugates and BCIP/NBT as described [13]. Line M displays a prestained molecular marker mixture.

As seen in Figure 4, bovine type II collagen possesses in the region of a 150 kDa, a protein with glutamine and lysine residues accessible for TGase (solid rectangle). The high molecular aggregates above 250 kDa could be small amounts of biotinylated, cross-linked collagen. The band at 38 kDa was caused by biotinylated TGase as result of auto-labeling (Figure 4, dotted rectangle). Similarly, the NP extract contains a glutamine donor protein migrating with similar velocity as bovine type II collagen, while the corresponding band of lysine labeling was absent or at best weak (Figure 4, solid rectangle). In contrast, a NP protein below 130 kDa, indicated by a broad band, seems to be a strong lysine and a weak glutamine donor (Figure 4, white rectangle). Moreover, a distinct band at about 55 kDa displays, undoubtedly, an additional protein containing reactive lysine residues (Figure 4, dashed rectangle). The deeply colored continuum in both lanes suggests the occurrence of additional TGase substrates in the NP extract, not forming distinct bands. The control consisting of NP extract and the biotinylated dipeptide (without TGase) showed weak bands, obviously caused by non-specific interaction between the hydrophobic probe and few NP proteins. Summarizing the results, porcine nucleus pulposus obviously contains several proteins capable of being cross-linked by bacterial TGase. For the development of gelatin-free NP hydrogels, these components have to be identified and produced recombinant. A mixture of such components can be used to form hydrogels cross-linked by TGase. Selective addition of TGase substrates could enhance cross-linking potential of NP extract hydrogels and would allow us to study their influence on hMSC differentiation towards NP cells.

## 4. Conclusions

In recent years, an increasing interest in the potential use of adult stem cells for IVD regeneration has emerged. Research focused on the impact of differentiation conditions and of biomaterials *in vitro* and *in vivo*. The current study investigated the influence of gelatin and NP extract on mesenchymal stem cell differentiation. Furthermore, the suitability of NP extract for cross-linking with TGase was examined to use this extract as substrate for hydrogel formation.

In pure gelatin, hMSC-TERT did not differentiate into NP-like cells. Although differentiation conditions were the same as for the experiments in agarose [6], the developed cell phenotype differed completely. In all investigated approaches, the microscopical and gene expression results indicated more a chondrocyte-like than a NP-like cell development. The putative NP cell marker PAX1 was the only gene specifically switched on or off between the different approaches. Further clarification is required to establish whether this gene could be a real NP cell marker or not. All other putative markers were present in all experimental groups. Thus, complementary quantitative measurements of RT-PCR will be necessary and will be performed in the future.

The type I collagen character of the used gelatin probably has a too strong an impact on the cells. It was notable that NP extract could direct the differentiation more into NP-like cells, but the cell phenotype still remains chondrogenic. Therefore, it would be of great interest to investigate pure NP extract as hydrogel material. As demonstrated in the current study, distinct proteins could be labeled by TGase mediated incorporation of biotinylated probes. This indicates the presence of both glutamine and lysine donor proteins. Porcine nucleus pulposus is obviously an appropriate material to construct 3-D scaffolds, stabilized by bacterial TGase, for the proliferation and differentiation of mesenchymal stem cells. We have started the process of partitioning and separating the components of the NP extract by chromatographic methods, in order to identify and characterize highly purified proteins using similarly specific antibodies and the TGase probes. In particular, the association behavior of the isolated proteins will be studied by microcalorimetric measurements. Finally, a hydrogel, only consisting of NP components, has to be formed and investigated in the future.

For a successful NP regeneration approach, it is imperative to implant absolutely correct differentiated cells. The implantation of chondrocyte-like cells, which do not have the correct NP cell phenotype, can result in the formation of a tissue that does not mimic the highly hydrated NP. These tissues develop other morphologic or biomechanical properties, and do not restore a functional IVD [39]. To overcome this problem, an appropriate NP cell marker has to be identified first. Non-human studies are less suitable for this identification because the expression level of the genes differs too much between different species. Moreover, not only NP cells and chondrocytes have to be compared. The expression of promising markers also has to be analyzed in MSCs and notochordal cells. Secondly, when using stem cells, differentiation conditions and biomaterials have to be investigated to guarantee the presence and maintenance of the right phenotype.
